# Advanced Biomaterials and Technologies in Implantology

**DOI:** 10.1155/2012/302825

**Published:** 2012-12-25

**Authors:** Yasuhiro Tanimoto, Yo Shibata, Jukka Pekka Matinlinna, Osamu Komiyama, Masaru Yamaguchi

**Affiliations:** ^1^Department of Dental Biomaterials, Nihon University School of Dentistry at Matsudo, 2-870-1 Sakaecho Nishi, Chiba, Matsudo 271-8587, Japan; ^2^Department of Oral Biomaterials and Technology, Showa University School of Dentistry, Tokyo 142-8555, Japan; ^3^Department of Dental Materials Science, Faculty of Dentistry, The University of Hong Kong, Sai Ying Pun, Hong Kong; ^4^Department of Clinical Oral Physiology, Nihon University School of Dentistry at Matsudo, Chiba, Matsudo 271-8587, Japan; ^5^Department of Orthodontics, Nihon University School of Dentistry at Matsudo, Chiba, Matsudo 271-8587, Japan

The variety of implant devices has been available because modifications in design or materials will enable superior performance of the implant. Events leading to integration of an implant into bone, and hence determining the performance of a device, take place largely at the tissue and implant interface. These include not only implant-related factors, such as material shape, topography, and surface chemistry, but mechanical loading, surgical techniques, and host immunity as well. This special issue focuses on both materials science and clinical practice in the current implantology, to improve the quality and longevity of human life. For example, in implantology based on materials science, an understanding of the bone-implant interface is critical for the development of proactive titanium implants that can promote desired outcomes. Especially, the ideal nanomicroscale topography and chemistry should be defined for enhancing implant integration into native tissue. Meanwhile, in implantology based on clinical practice, an understanding of the dynamics and anatomical and biological concepts of the periodontium and peri-implant tissues both at the surgical and prosthetic phases of treatment which contributes to better soft and hard tissue management is important, for implant specialists.

With the above-described points based on both materials science and clinical practice as background, this special issue contains five research papers and four review papers to contribute better understanding and solution involved in the current implantology as follows.

In the paper entitled *“The effect of zirconia in hydroxyapatite on Staphylococcus epidermidis growth,”* W. Siswomihardjo et al. investigate the effect of zirconia concentrations in hydroxyapatite on the growth of *Staphlococcus epidermidis*. Recently, many researchers have reported with regard to both mechanical and biological properties of composites of hydroxyapatite and inert high-strength bioceramics such as zirconia and alumina. They report that local hydroxyapatite with 20% zirconia proved to be an effective concentration to inhibit the growth of *Staphlococcus epidermidis* colony.

In the paper entitled *“Cytotoxicity of Cricula triphenestrata cocoon extract on human fibroblasts,”* S. Sunarintyas et al. present the cytotoxicity of Indonesian silkworm cocoon extract of *Cricula triphenestrata* on human fibroblasts. They indicate that *Cricula triphenestrata* cocoon extract has the potential for the use of bone substitutes, because that is not cytotoxic on human gingival fibroblast cells.

In the paper entitled *“Stress analysis of a class II MO-restored tooth using a 3D CT-based finite element model,”* Y. P. Chan et al. propose a method to create a 3D finite element assembly model for a restored tooth and its corresponding mandible based on CT images. In this study, the tooth model is a triphasic one, consisting of enamel, dentin, and pulp phases, which gives rise to a more realistic simulation to estimate the stress distribution of the tooth and the restoration. The proposed method offers a computational scheme assisting dentists to design a more effective tooth repair strategy.

In the paper entitled *“Soft and hard tissue management in implant therapy—part I: surgical concepts,”* A. D'Addona et al. discuss the reason why surgical augmentation procedures are often required to enhance postextraction sites. Moreover, they discuss the use and selection of autogenous grafts, nonautogenous graft material, and finally the timing of implant placement in relation to the extraction, which have been identified as the key concepts in the soft and hard tissue management (SHTM).

In the paper entitled *“Soft and hard tissue management in implant therapy—part II: prosthetic concepts,”* P. H. Manicone et al. describe the key concepts both the theoretical and clinical prosthetic components which the literature has emphasized as having an important role in SHTM in implant therapy, by discussing their direct effect on these structures, and hence, on the final result.

In the paper entitled *“Assessment of the quality of newly formed bone around titanium alloy implants by using X-ray photoelectron spectroscopy,”* H. Nakada et al. analyze the differences in bones quality between newly formed bone and cortical bone formed around titanium alloy implant by using X-ray photoelectron spectroscopy (XPS). XPS analysis is a useful technique for determining of information on elemental qualitative and quantitative bone composition. This paper presents that the peaks and quantities of each element of newly formed bone were similar to those of cortical bone at 8 weeks, suggestive of a strong physicochemical resemblance.

In the paper entitled *“Mini-implants in the anchorage armamentarium: new paradigms in the orthodontics,”* M. Yamaguchi et al. present the development, clinical use, benefits, and drawbacks of the miniscrew and plate type implants used to obtain a temporary but absolute skeletal anchorage for orthodontic applications. As a future of mini-implants, the application of calcium phosphate-based biomaterials as new types of mini-implants is proposed, because the improvement in designed screw including the diameter, length, and thread may reach the limit.

In the paper entitled *“Clinical management of implant prostheses in patients with bruxism,”* O. Komiyama et al. discuss the importance of the occlusal scheme used in implant restorations for implant longevity, and suggest a clinical approach and occlusal materials for implant prostheses in order to prevent complications related to bruxism. As is well known, dental implant must withstand varying degrees of force during mastication and for some patients from bruxism or clenching. The authors therefore explain that the clinical management of bruxism will become an important subject for implant prostheses.

In the paper entitled *“MC3T3-E1 cells on titanium surfaces with nanometer smoothness and fibronectin immobilization,”* T. Hayakawa et al. investigate the effects of mechanical treatment (nanometer smoothing and sandblasting) and biochemical treatment (with and without fibronectin immobilization) of a titanium surface on cell viability and total protein contents of MC3T3-E1 on the titanium surface. Fibronectin can be easily immobilized on both nanometer smooth and sandblasted titanium surfaces by their originally developed method, namely, the tresyl chloride-activation technique. They report that the combination of sandblasting and fibronectin immobilization enhanced the cell viability and fibronectin immobilization providing better arrangements of attached cells.

Finally, it is hoped that the contents of this special issue might provide valuable insights, to the researcher and the clinician, into the important topic of implantology.

*Yasuhiro Tanimoto* *Yasuhiro Tanimoto* *Yo Shibata* *Yo Shibata* *Jukka Pekka Matinlinna* *Jukka Pekka Matinlinna* *Osamu Komiyama* *Osamu Komiyama* *Masaru Yamaguchi* *Masaru Yamaguchi*

